# Supplementary material for: Female copulation song is modulated by seminal fluid
Source: Nat Commun. 2020 Mar 18;11:1430. doi: 10.1038/s41467-020-15260-6 (PMC7080721; doi:10.1038/s41467-020-15260-6)
Supplement: Supplementary file 1 — Supplementary Information [file 41467_2020_15260_MOESM1_ESM.pdf]

Supplementary Information for

Female copulation song is modulated by seminal fluid

Kerwin et al.

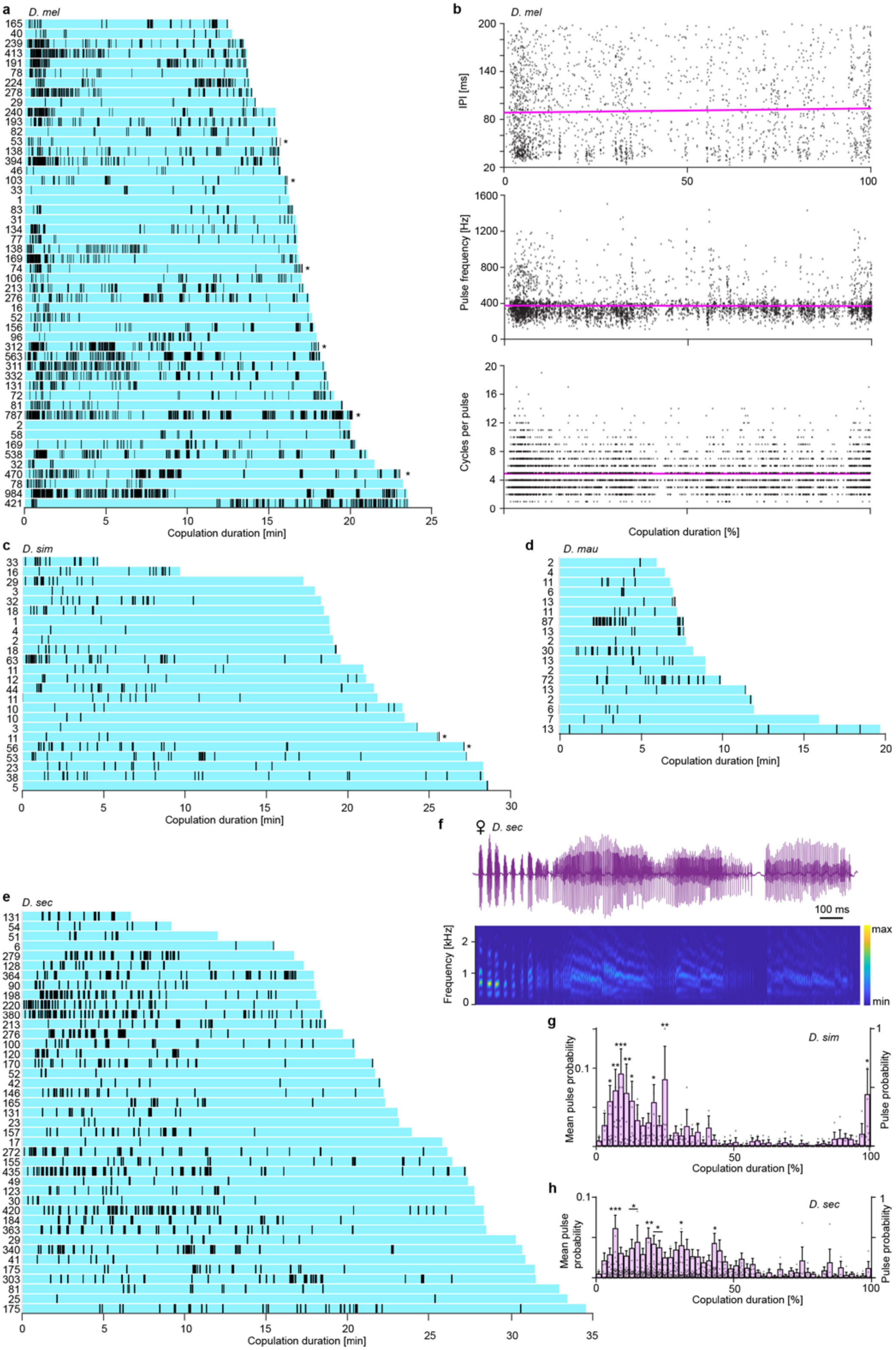
a
D. mel
Copulation duration [min]
0
5
10
15
20
25
b
D. mel
IPI [ms]
200
140
80
20
0
50
100
Pulse frequency [Hz]
1600
1200
800
400
0
Cycles per pulse
20
16
12
8
4
0
Copulation duration [%]
c
D. sim
Copulation duration [min]
30
d
D. mau
Copulation duration [min]
e
D. sec
Copulation duration [min]
35
f
♀ D. sec
100 ms
Frequency [kHz]
2
1
0
max
min
g
D. sim
Mean pulse probability
0.1
Pulse probability
1
Copulation duration [%]
h
D. sec
Mean pulse probability
0.1
Pulse probability
1
Copulation duration [%]

Supplementary Figure 1

Female copulation song occurs throughout copulation

a Raster plots of *D. melanogaster* female song pulses from 50 of the 92 copulations evaluated in Figure 1c, ordered by copulation duration. Cyan indicates copula, song pulses are in black. The number at the left of each line gives the total number of song pulses for each copulation. Stars at the end of lines indicate that few song pulses were detected immediately after disengagement of the couple. **b** IPI, pulse frequency and cycles per pulse of copulation song pulses plotted against their time of occurrence during copulation. 4512 pulses from n = 43 copulations were pooled. Each data point represents one song pulse. Magenta lines depict linear regression lines: y = 0.05x + 88.5, R^2 = 0.001 (IPI); y = -0.05x + 377, R^2 = 0.00009 (frequency); y = -0.0001x + 4.92, R^2 = 0.000003 (cycles per pulse). **c - e** Raster plots of *D. simulans* (*D. sim*), *D. mauritiana* (*D. mau*) and *D. sechellia* (*D. sech)* female song pulses, displayed as in **a**. **f** Exemplary *D. sechellia* copulation song pulses with spectrogram. **g – h** Mean probability of female song pulses (magenta bars depicting 2% bins of total copulation duration, left y axis), and pulse probability of individual flies in 2% bins of total copulation duration (grey data points, right y axis) for *D. simulans* (*D. sim*, n = 24 copulations) and *D. sechellia* (*D. sech*, n = 41 copulations) throughout copulation, data from copulations displayed in **c, e**. Error bars indicate mean and s.e.m., p = 0.0224 (bin 3), p = 0.0061 (bin 4), p = 0.0006 (bin 5), p = 0.0087 (bin 6), p = 0.0224 (bin 7), p = 0.0258 (bin 11), p = 0.0016 (bin 13), p = 0.0104 (bin 50) in **g**, p = 0.00047 (bin 4), p = 0.046 (bin 7) p = 0.0136 (bin 8), p = 0.0057 (bin 10), p = 0.0199 (bin 11), p = 0.0418 (bin 12), p =0.0262 (bin 16), p = 0.0169 (bin 22) in **h**, permutation test (one-sided). Source data are provided as a Source data file.

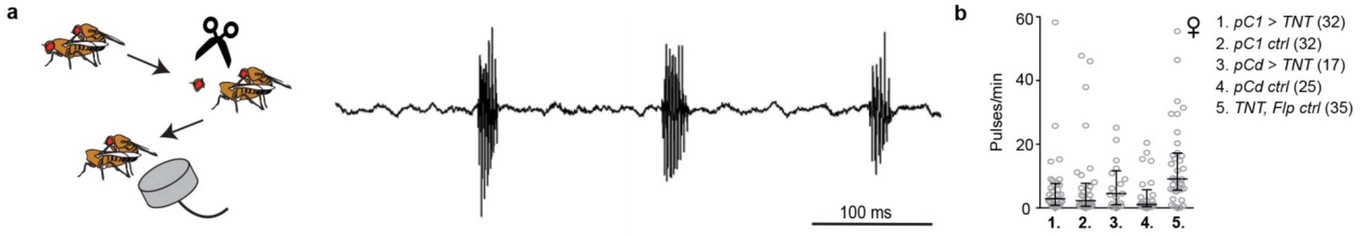

Supplementary Figure 2

Brain neurons are not required for copulation singing

a Females are beheaded with spring scissors immediately after copulation initiation and song is recorded afterwards. Exemplary copulation song pulses from a beheaded *CS wt* female. **b** Song pulses per minute copulation (with *CS wt* males) for females with the *dsx+* brain neuronal classes pC1 and pCd silenced with tetanus toxin (TNT) and controls. For full genotypes, see Methods. Each data point represents one fly, n is indicated after the genotypes, error bars indicate median and interquartile range, no significant difference, Kruskal-Wallis test with Dunn's multiple comparison (two-sided). Source data are provided as a Source data file.

**Virgin genotype**	**n tested**	**n copulated**	**Copulation rate [%]**
w; UAS-TNT, UAS-CD8-Gfp/ Otd-nlsFLPo; tubP-FRT-GAL80-FRT/ dsx-GAL4	32	0	0
w; UAS-FRT-stop-FRT-TNT / Otd-nlsFLPo; dsx-GAL4	125	5	4
w; UAS-TNT, UAS-CD8-Gfp/ tsh-GAL80; dsx-GAL4	47	0	0

Supplementary Table 1

Silencing of all *dsx+* brain neurons leads to very low copulation rates

Copulation rates (after pairing with a *CS wt* male for 40 min) for virgin females (three different genotypes) with all *dsx+* brain neurons silenced.

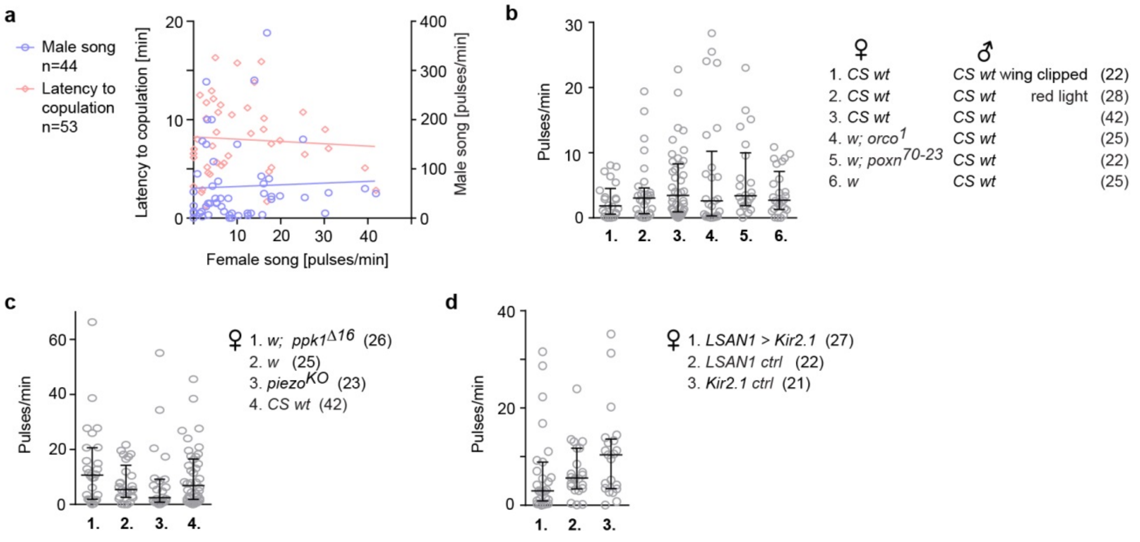

Supplementary Figure 3

Precopulatory courtship and mechanosensation do not influence copulation singing

a Amount of male precopulatory courtship song (blue, right y axis) and latency to copulation (red, left y axis) plotted against amount of female song. Each data point represents one *CS wt* couple. p = 0.61 and p = 0.33, respectively, Spearman, no significant correlation. **b – d** Song pulses per minute copulation for females from couples with indicated genotypes/conditions. **b** Manipulation of sensory input relevant for courtship. **c** Manipulation of female mechanosensors expressed in the reproductive tract. **d** Silencing of LSAN neurons. For full genotypes, see Methods. Each data point represents one fly, n is indicated after the genotypes/conditions, error bars indicate median and interquartile range, no significant difference, Mann-Whitney test or Kruskal-Wallis test with Dunn's multiple comparison (two-sided). Source data are provided as a Source data file.

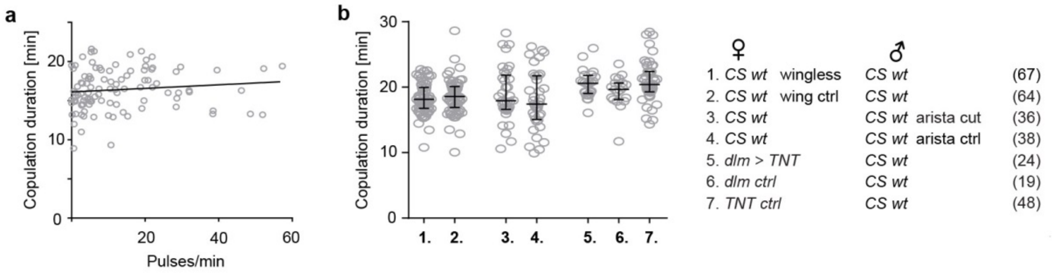

Supplementary Figure 4

Female song does not affect copulation duration

a Copulation duration of n = 103 *CS wt* couples plotted against the amount of female sound. **b** Copulation duration of couples with indicated genotypes/conditions. Each data point represents one couple, n is indicated after the genotypes/conditions, error bars indicate median and interquartile range, no significant difference, Mann-Whitney test or Kruskal-Wallis test with Dunn's multiple comparison (two-sided). Source data are provided as a Source data file.

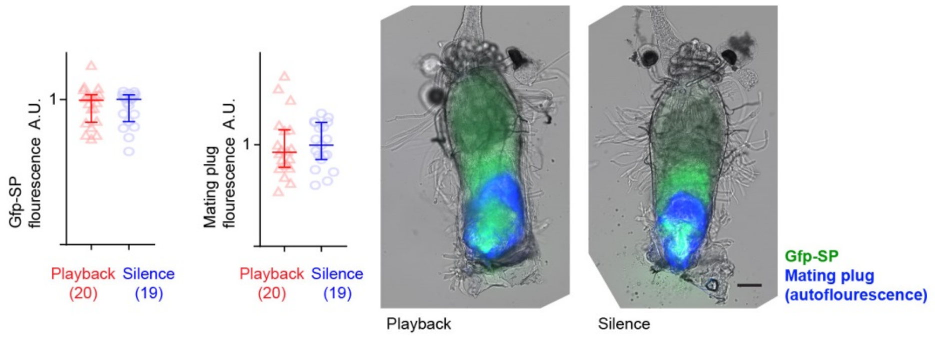

Supplementary Figure 5

Transfer of sex peptide and mating plug in response to copulation song playback

Flourescence intensity of SP-Gfp and autoflourescence of the mating plug in the bursa of mute (*dlm > TNT*) females after copulations supplemented with female song playback (red) or occurring in silence (blue). Each data point represents one female, n is indicated after the conditions, error bars indicate median and interquartile range, no significant difference, Mann-Whitney test (two-sided). Representative bursae from the two conditions, with SP-Gfp in green and mating plug in blue. Scale bar, 100 µm. Source data are provided as a Source data file.
