# Supplementary material for: Female copulation song is modulated by seminal fluid
Source: Nat Commun. 2020 Mar 18;11:1430. doi: 10.1038/s41467-020-15260-6 (PMC7080721; doi:10.1038/s41467-020-15260-6)
Supplement: Supplementary file 3 — Reporting Summary [file 41467_2020_15260_MOESM3_ESM.pdf]

nature research

Corresponding author(s): Anne C. von Philipsborn

Last updated by author(s): Feb 18, 2020

Reporting Summary

Nature Research wishes to improve the reproducibility of the work that we publish. This form provides structure for consistency and transparency in reporting. For further information on Nature Research policies, see Authors & Referees and the Editorial Policy Checklist .

Statistics

For all statistical analyses, confirm that the following items are present in the figure legend, table legend, main text, or Methods section.

n/a	Confirmed	
☐	☒	The exact sample size (*n*) for each experimental group/condition, given as a discrete number and unit of measurement
☐	☒	A statement on whether measurements were taken from distinct samples or whether the same sample was measured repeatedly
☐	☒	The statistical test(s) used AND whether they are one- or two-sided<br>*Only common tests should be described solely by name; describe more complex techniques in the Methods section.*
☒	☐	A description of all covariates tested
☐	☒	A description of any assumptions or corrections, such as tests of normality and adjustment for multiple comparisons
☐	☒	A full description of the statistical parameters including central tendency (e.g. means) or other basic estimates (e.g. regression coefficient) AND variation (e.g. standard deviation) or associated estimates of uncertainty (e.g. confidence intervals)
☐	☒	For null hypothesis testing, the test statistic (e.g. *F*, *t*, *r*) with confidence intervals, effect sizes, degrees of freedom and *P* value noted<br>*Give P values as exact values whenever suitable.*
☒	☐	For Bayesian analysis, information on the choice of priors and Markov chain Monte Carlo settings
☒	☐	For hierarchical and complex designs, identification of the appropriate level for tests and full reporting of outcomes
☒	☐	Estimates of effect sizes (e.g. Cohen's *d*, Pearson's *r*), indicating how they were calculated

Our web collection on statistics for biologists contains articles on many of the points above.

Software and code

Policy information about availability of computer code

Data collection	Imaging data: Zeiss Zen 2012 software<br>Audio recording: National Instruments software supporting NI USB-6259 MASS Term, custom written MATLAB R2015b scripts
Data analysis	custom written MATLAB R2015b (Mathworks) scripts, male courtship song analysis code: FlySongSegmenter, by B. Arthur and S. Stern (deposited at https://github.com/FlyCourtship/FlySongSegmenter) GraphPad software Prism6, Fiji/ImageJ, Zeiss Zen Blue 2012

For manuscripts utilizing custom algorithms or software that are central to the research but not yet described in published literature, software must be made available to editors/reviewers. We strongly encourage code deposition in a community repository (e.g. GitHub). See the Nature Research guidelines for submitting code & software for further information.

Data

Policy information about availability of data

All manuscripts must include a data availability statement. This statement should provide the following information, where applicable:

- Accession codes, unique identifiers, or web links for publicly available datasets
- A list of figures that have associated raw data
- A description of any restrictions on data availability

The datasets generated during the current study are available from the corresponding author on reasonable request. All figures have associated raw data.

Field-specific reporting

Please select the one below that is the best fit for your research. If you are not sure, read the appropriate sections before making your selection.

Life sciences study design

All studies must disclose on these points even when the disclosure is negative.

Sample size	No sample size calculations were performed. Sample size was determined by either experimental feasibility or based on previous studies in the field (for fly song parameters, see e.g. Shirangi et al. 2016, ref. 9; O'Sullivan et al. 2018, ref 28, for sperm count in female storage organs e.g. Garbaczewska et al. 2013, ref. 25; Lüpold et al. 2011, ref. 23; for female remating percentages and latencies e.g. Shao et al. 2019, ref. 38; Zhou et al. 2014, ref. 30.)<br>Sample size was estimated to be appropriate and sufficient based on consistency of distributions and differences between groups (genetic or experimental conditions).
Data exclusions	No data were excluded from analysis.
Replication	All experiments were replicated at least once independently (performed on different days, using flies from different batches or independent genetic crosses) and all attempts at replication were successful.
Randomization	Random allocation of flies of the same genotype to different experimental groups was performed by randomly aspirating flies from culture vials.
Blinding	During data analysis (annotation of song pulses) investigators were blinded to group allocation.

Reporting for specific materials, systems and methods

We require information from authors about some types of materials, experimental systems and methods used in many studies. Here, indicate whether each material, system or method listed is relevant to your study. If you are not sure if a list item applies to your research, read the appropriate section before selecting a response.

Materials & experimental systems

n/a	Involved in the study	
☐	☒	Antibodies
☒	☐	Eukaryotic cell lines
☒	☐	Palaeontology
☐	☒	Animals and other organisms
☒	☐	Human research participants
☒	☐	Clinical data

Methods

n/a	Involved in the study	
☒	☐	ChIP-seq
☒	☐	Flow cytometry
☒	☐	MRI-based neuroimaging

Antibodies

Antibodies used	anti-GFP antibody (rabbit), Torrey Pines Biolabs, Cat# TP401 071519; RRID:AB_10013661,<br>nc82/ bruchpilot antibody (mouse), Developmental Studies Hybridoma Bank, Cat# nc82; RRID:AB_2314866<br>secondary antibodies:<br>Goat anti-Mouse IgG Alexa Fluor 647 and Goat anti-Rabbit IgG Alexa Fluor 488 (Thermo Fisher Scientific, Cat# A-21236; RRID: AB_2535805 and Cat# A-11034; RRID: AB_2576217).
Validation	anti-GFP antibody (rabbit), Torrey Pines Biolabs: for validation see entry at antibodyregistry.org: RRID:AB_10013661<br>nc82/ bruchpilot antibody (mouse), Developmental Studies Hybridoma Bank: for validation see entry at antibodyregistry.org:AB_2314866 or validation notes at providers website: https://dshb.biology.uiowa.edu/nc82<br>These antibodies are standard in the field, see also method section for relevant references, e.g. O'Sullivan et al. 2018, Curr Biol. 28 (reference 28).

Animals and other organisms

Policy information about studies involving animals; ARRIVE guidelines recommended for reporting animal research

Laboratory animals	Drosophila melanogaster, Drosophila simulans, Drosophila mauritiana, Drosophila sechellia, wild type laboratory strains and transgenic strains, genotype, age and sex specified in detail in the methods.
Wild animals	The study did not involve wild animals.
Field-collected samples	The study did not involve field-collected smaples.
Ethics oversight	No regulations apply for the invertebrate Drosophila.

Note that full information on the approval of the study protocol must also be provided in the manuscript.
