# Supplementary material for: Female copulation song is modulated by seminal fluid
Source: Nat Commun. 2020 Mar 18;11:1430. doi: 10.1038/s41467-020-15260-6 (PMC7080721; doi:10.1038/s41467-020-15260-6)
Supplement: Supplementary file 4 — Description of Additional Supplementary Files [file 41467_2020_15260_MOESM4_ESM.pdf]

Description of Additional Supplementary Files

File Name: Supplementary Audio 1
Description: **Female copulation song**
Representative sound recording of female copulation song, sampling rate 10,000.

File Name: Supplementary Movie 1
Description: **Female wing vibration during copulation song**
High-speed video (2,000 frames/s) played 100x slowed down (20 frames/s) of a female producing copulation song pulses by bilateral wing vibration and corresponding sound oscillogram.
